# Interim Report on the Examination of Corrosion Damage in Homes Constructed With Imported Wallboard: Examination of Samples Received September 28, 2009

**DOI:** 10.6028/jres.115.012

**Published:** 2010-06-01

**Authors:** D. J. Pitchure, R. E. Ricker, M. E. Williams, S. A. Claggett

**Affiliations:** Metallurgy Division, Materials Science and Engineering Laboratory, National Institute of Standards and Technology, Gaithersburg, MD 20899-8553

**Keywords:** atmospheric corrosion, copper, copper sulfide, household appliances, hydrogen sulfide, indoor atmosphere, sulfide, wallboard

## Abstract

Since many household systems are fabricated out of metallic materials, changes to the household environment that accelerate corrosion rates will increase the frequency of failures in these systems. Recently, it has been reported that homes constructed with imported wallboard have increased failure rates in appliances, air conditioner heat exchanger coils, and visible corrosion on electrical wiring and other metal components. At the request of the Consumer Product Safety Commission (CPSC), the National Institute of Standards and Technology (NIST) became involved through the Interagency Agreement CPSC-1-09-0023 to perform metallurgical analyses on samples and corrosion products removed from homes constructed using imported wallboard. This document reports on the analysis of the first group of samples received by NIST from CPSC.

The samples received by NIST on September 28, 2009 consisted of copper tubing for supplying natural gas and two air conditioner heat exchanger coils. The examinations performed by NIST consisted of photography, metallurgical cross-sectioning, optical microscopy, scanning electron microscopy (SEM), and x-ray diffraction (XRD). Leak tests were also performed on the air conditioner heat exchanger coils. The objective of these examinations was to determine extent and nature of the corrosive attack, the chemical composition of the corrosion product, and the potential chemical reactions or environmental species responsible for accelerated corrosion.

A thin black corrosion product was found on samples of the copper tubing. The XRD analysis of this layer indicated that this corrosion product was a copper sulfide phase and the diffraction peaks corresponded with those for the mineral digenite (Cu_9_S_5_). Corrosion products were also observed on other types of metals in the air conditioner coils where condensation would frequently wet the metals. The thickness of the corrosion product layer on a copper natural gas supply pipe with a wall thickness of 1.2 mm ± 0.2 mm was between 5 μm and 10 μm.

These results indicate that a chemical compound that contains reduced sulfur, such as hydrogen sulfide (H_2_S), is present in the environment to which these samples were exposed. The literature indicates that these species strongly influence corrosion rates of most metals and alloys even at low concentrations. None of the samples examined were failed components, and no evidence of imminent failure was found on any of the samples examined.

All of the corrosion damage observed to date is consistent with a general attack form of corrosion that will progress in a uniform and relatively predictable manner. No evidence of localized attack was found, but these forms of attack typically require an incubation period before they initiate. Therefore, the number of samples examined to date is too small to draw a conclusion on the relative probability of these forms of corrosion being able to cause or not cause failure. Samples from failed systems or from laboratory tests conducted over a wide range of metallurgical and environmental conditions will be required to assess the probability of these other forms of corrosion causing failure.

## 1. Introduction

Metallic materials in home construction are subject to corrosive attack by the normal internal atmosphere of homes as well as the other environments they encounter during service in the home. The corrosion resistance of the metals in these environments is a major factor in determining the normal deterioration of the performance of household appliances, switches, safety systems, and water, electrical, and gas supply systems. When these systems deteriorate more rapidly than normal, there is a high probability that accelerated corrosion is the cause. Identification of the factors responsible for accelerated corrosion can be done through three ways: (i) identification of the unique characteristics of the homes with accelerated deterioration, (ii) identification of the differences in the environments encountered in the homes with accelerated deterioration, or (iii) identification of failure modes, corrosion product compositions, and responsible chemical reactions.

Of immediate concern is the accelerated deterioration of systems in homes constructed with imported wallboard. Due to the very large number of complaints from the owners of homes constructed with imported wallboard, the Consumer Product Safety Commission (CPSC) has become concerned that this product contains chemical species that alter the internal atmosphere of the home and accelerate corrosion and the deterioration of systems that rely on metals to perform critical functions. As a result, the CPSC has instituted a large multitasked program to look at this product and how it influences the health of occupants, the performance of critical household systems, and the prevalence of this product in homes in the US. As part of this program, the Materials Science and Engineering Laboratory (MSEL) of the National Institute of Standards and Technology (NIST) became involved through the Interagency Agreement CPSC-1-09-0023 to examine metallic samples removed from service in homes constructed using imported wallboard. The objective of these examinations is to identify potential corrosion related failure modes, corrosion product chemistry, and potential chemical reactions. This document reports on the findings of the examinations performed on the first group of samples provided to NIST by CPSC. All findings reported here should be considered preliminary, as more examinations are planned. None of the samples provided to NIST in the first group were failed components. Therefore, none of the conclusions drawn here can address the mechanisms of the reported failures.

## 2. Examinations

### A. Sample Selection and Delivery

The samples were selected by CPSC staff and transported and hand delivered to NIST. The samples were received on September 28, 2009 and immediately cataloged for recording and tracking the examinations. All of the samples received were identified with a number assigned to the sample by CPSC. These numbers consisted of eleven digits, starting with: 09-302-####-##. NIST used the last six unique digits of the samples to track them during the examinations. The samples received consisted of a number of lengths of copper tubing used as natural gas supply lines and two air conditioner heat exchanger coils.

### B. Visual Examination and Photography

**Fig. 1:**
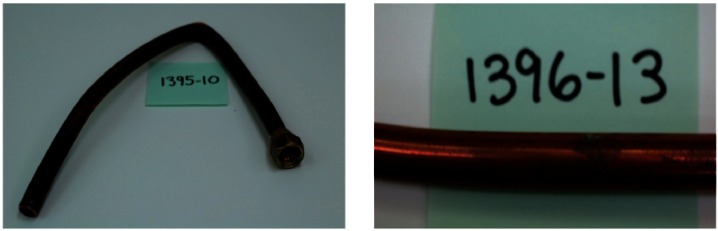
Photographs of two representative samples of copper natural gas supply tubing.

**Fig. 2:**
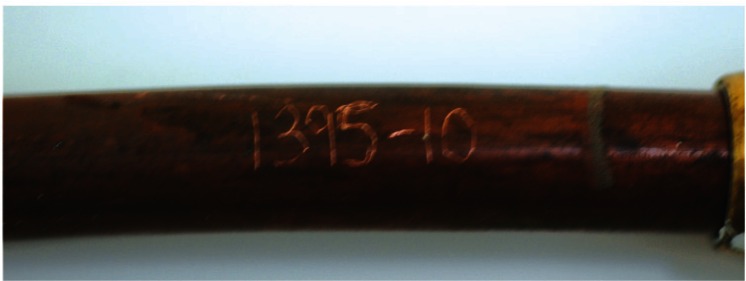
Scribe marks illustrating the contrast between the color of bare copper and a sample of copper natural gas line tubing.

The lengths of copper tubing were discolored to varying degrees. This discoloration, which is illustrated in Fig. 1, was found to be due to the presence of a thin adherent layer of a black substance. This is clearly illustrated in Fig. 2 where the sample number has been scribed into the sample exposing fresh bare copper. The contrast in this figure is obvious. While this film adhered to the surface, physical contact would result in the transfer of a small quantity of a fine black powder from the film. In some cases, the discoloration was found to be very uniform, but in other cases it was irregular. The extent of discoloration varied with the thickness of the layer.

**Fig. 3:**
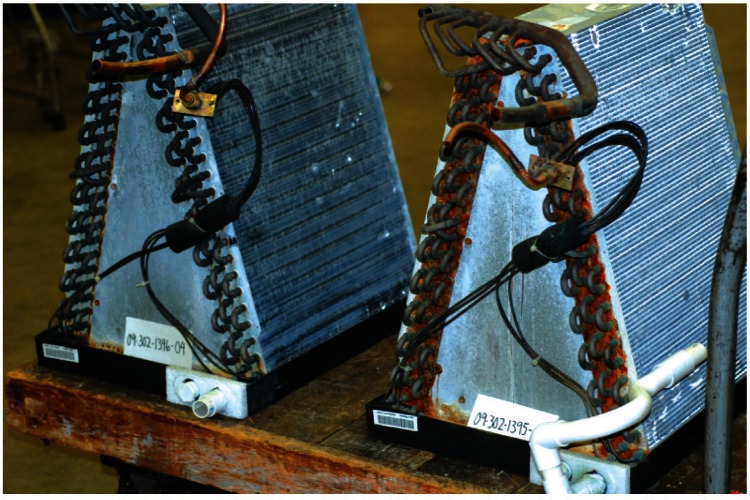
Photograph of the two air conditioner evaporator coils showing the black discoloration of the copper U-bends and corrosion of the end plate.

**Fig. 4:**
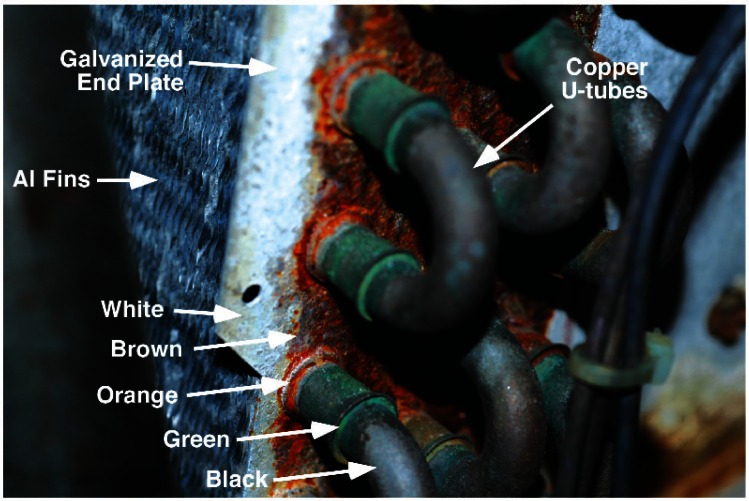
Close-up photograph of the U-tubes and end plate of an air conditioner evaporator coil showing that there appears to be 5 colors associated with the corrosion products: white, brown, orange, green, and black.

The air conditioner evaporator coils were also examined, Fig. 3. From the two coils in this figure it can be seen that all exposed copper surfaces have turned dark. These coils are constructed out of parallel lines of copper tubing placed through thin sheets of aluminum that serve as the heat transfer fins. The copper tubing is linked together by soldered U-bends to the ends of adjacent lengths of copper tubing. The end plates that hold this assembly in place are made out of galvanized steel. Copper manifolds and valves complete the assembly. Figure 4 is a close-up of the end plates and the U-bend region. In this figure, brown and orange corrosion products can be seen on the galvanized end plates, while black and green corrosion products can be seen on the copper tubing. The normal patina that forms on copper in the cyclically wet and dry environment of an air conditioner evaporator coil would be expected to contain copper (I) or copper (II) ions in oxides (black to brownish-red), carbonates (green to blue), sulfates (bright blue), and chlorides (greenish white to blue-green) [1,2]. Again, the black color was found to be due to the presence of a thin layer of a loose black powder.

### C. Identification of the Black Corrosion Product by X-Ray Diffraction

**Fig. 5:**
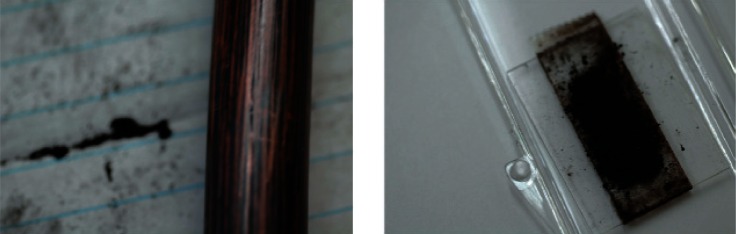
Photographs of the scraped copper natural gas supply line and the slide holding the sample for x-ray diffraction.

The black substance observed on the surfaces of the copper components was identified by x-ray diffraction. This was accomplished by scraping the black substance from the surface of one of the sample of copper natural gas line tubing using a razor blade. The powder removed in this manner was accumulated on double stick tape and attached to a glass slide as shown in Fig. 5. A powder diffraction pattern was then obtained from this sample of scrapings using Siemens D500 x-ray diffractometer with a Cu K*_a_* radiation source operated at 40 kV and 30 mA using a step size of 0.03 degrees, and a dwell time of 5 s.^1^

**Fig. 6:**
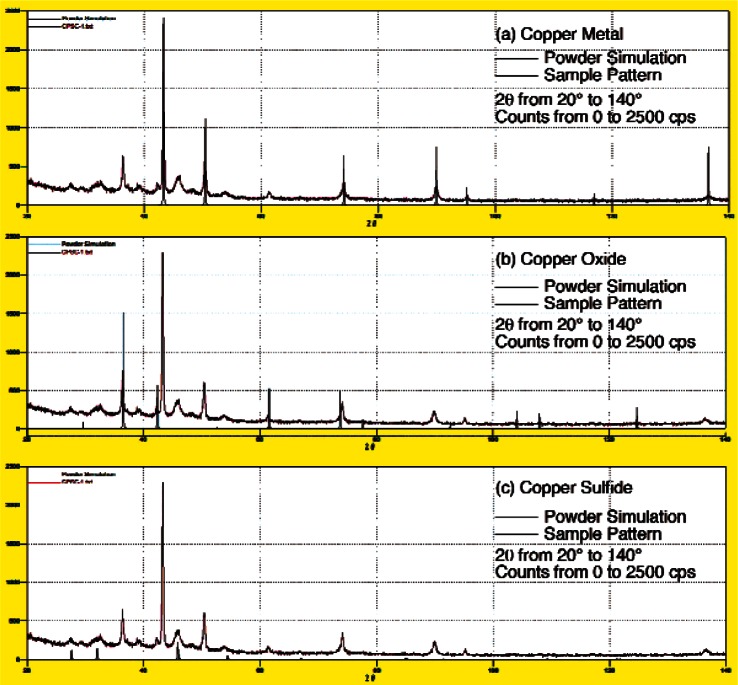
Powder diffraction patterns for the sample of corrosion products scraped off a copper natural gas supply line (red) with, identified in black, the peaks for (a) copper, (b) copper (I) oxide (ICSD #38233), and (c) copper (I) sulfide (ICSD #95395).

**Fig. 7:**
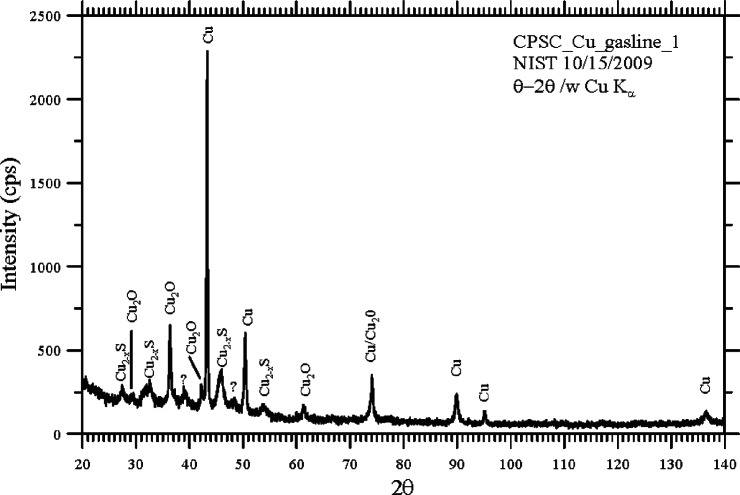
Summary of peaks found in the corrosion product sample and their identification for showing that only two small peaks were unidentified by the three phases.

Figure 6 shows the results of these measurements in red along with the peak locations in black for copper metal (Fig. 6a), copper (I) oxide (Fig. 6b) and copper (I) sulfide (Fig. 6c) as listed in the International Crystal Structure Database (ICSD). This figure shows that peaks corresponding to all three of these phases are present in the sample of scrapings from the surface of the copper natural gas supply line. Given that the sample was collected by scraping the surface of copper metal with a sharp razor blade, the peaks for copper and copper oxide were expected since copper is a soft metal compared to the steel of the razor blade and will normally form copper oxide when exposed to air. The third phase, copper sulfide (Cu_2_S) is not a typical atmospheric corrosion product as the concentration of the reduced sulfur species, such as H_2_S, required to form this product is usually very low under normal atmospheric conditions [1, 3–5]. Figure 7 is a summary of the XRD results showing that all but two small peaks, indicated by question marks, are accounted for by these three phases.

### D. Air Conditioner Evaporator Coil Leak Testing

Leak testing was performed on the air conditioner heat exchange coils by attaching air tight fittings to the ends of the copper lines and connecting these to a helium gas detector. The inside of the coil was evacuated and helium gas passed over different regions of the coils. No leaks were found by this method. Then, the coils were pressurized with helium and a solution of soapy water was squirted onto different regions of the coils. This method also failed to find any leaks in these coils.

### E. Air Conditioner Evaporator Coil Sectioning

**Fig. 8:**
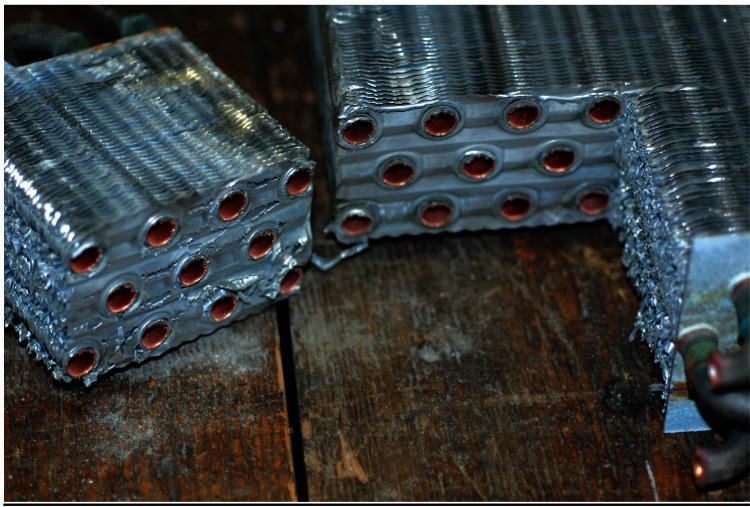
Air conditioner evaporator coil after slicing to allow for examination for corrosion damage between the aluminum sheets and the copper tubing.

To evaluate the possibility of corrosion damage occurring between the aluminum heat transfer fins and the copper tubing inside them, the heat exchanger unit was sliced with a band saw as shown in Fig. 8 to expose these internal surfaces for examination of the copper. No evidence of localized attack was found, and the black corrosion product layer was not detected by visual examination.

### F. Measurement of Corrosion Product Layer Thickness

**Fig. 9:**
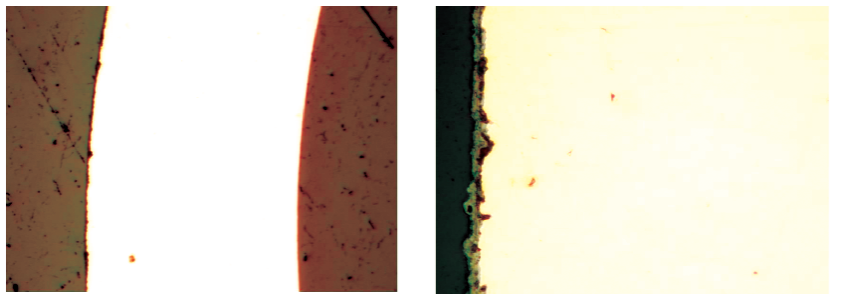
Optical micrographs of the wall of a copper natural gas supply line and the corrosion product layer at two magnifications (wall ≈1.2 mm thick).

The thickness of the black corrosion product was measured using optical microscopy of samples prepared in cross section. For this analysis, samples of natural gas supply lines were cut, mounted in epoxy, and polished using standard metallurgical practice, exercising care to maintain the edge of the sample with the corrosion products in place. Figure 9 shows optical micrographs of this sample where the corrosion product layer varied from 5 μm to 10 μm thick.

## 3. Discussion and Conclusions

The copper metal surfaces of the samples provided for examination were covered with a black film that appears to be a corrosion product. The x-ray powder diffraction results unambiguously identified this substance as a copper sulfide phase. Copper sulfides (Cu*_x_*S*_y_*) can have a wide range of stoichiometry ranging from CuS_2_ to Cu_2_S with 9 distinctly different phases having been identified over this range depending on the stoichiometry. The powder diffraction pattern was consistent with the digenite phase of copper sulfide that has a stoichiometry of Cu_9_S_5_ or Cu_1.8_S. The presence of a copper sulfide phase on the surface of the copper indicates that reduced sulfur compounds, such as hydrogen sulfide (H_2_S), sulfur (S_8_), or mercaptans (thiols), are present in the indoor atmosphere of the homes from which these samples were removed.

Reduced sulfur compounds generally accelerate the corrosion of engineering metals, as sulfur forms stable compounds with most of the parent and alloying elements in these alloys [3]. However, copper and silver are reported to have the greatest sensitivity to this environmental contaminant [2,6]. Rice et al. [2,6] conducted studies on the sensitivity of indoor corrosion rates of different metals to environmental contaminants including hydrogen sulfide. These authors fit the rate of mass increase (*dm*/*dt*) to the Eq. (1)

(1) $$\left(\frac{dm}{dt}\right)=A{\left[H_{2}S\right]}^{n}$$

where *A* is a constant, [H_2_S] is the concentration or activity of hydrogen sulfide in the atmosphere and *n* is a measure of the sensitivity of the corrosion rate to changes in the hydrogen sulfide concentration. This parameter, *n*, is also the slope of a log-log plot as

(2) $$\log\left(\frac{dm}{dt}\right)=\log A+n\log[H_{2}S].$$

Table 1 shows the results of Rice et al. [2,6]. This table clearly shows that copper and silver are the most sensitive of these metals to the concentration of hydrogen sulfide.

While the results clearly indicate that a reduced sulfur species is present in the indoor atmosphere of the homes that these samples came from, the corrosion product layer observed on these samples was less than 10 μm thick. This layer makes the copper look dark due to the black color and opaque nature of this corrosion product. All of the corrosion damage observed to date is consistent with a general attack form of corrosion that will progress in a uniform and relatively predictable manner. No evidence of any type of localized attack was found, but these forms of attack typically require an incubation period before they initiate. Therefore, the number of samples examined to date is too small to draw a conclusion on these forms of corrosion being able to cause or not cause failure. Samples from failed systems or from laboratory tests conducted over a wide range of metallurgical and environmental conditions will be required to assess the probability of these other forms of corrosion causing failure.

## Figures and Tables

**Table 1:** The corrosion rate parameter, *n*, in equations (1) and (2) for different metals exposed to hydrogen sulfide at different concentrations in air specifically formulated to represent a typical indoor air environment and air that is purified to remove all contaminants other than hydrogen sulfide.[Rice, 1982 #4]

Metal	Cu	Ag	Co	Ni	Fe
Representative Indoor Air	0.30	0.55	0.02	0.03	0.05
Purified 70 % RH Air	0.93	0.14	—	—	—

## Footnotes

1 Certain commercial equipment, instruments, or materials are identified in this paper to foster understanding. Such identification does not imply recommendation or endorsement by the National Institute of Standards and Technology, nor does it imply that the materials or equipment identified are necessarily the best available for the purpose.
